# *Cirsium arvense* and *Cirsium vulgare*: Comparative Ethnopharmacology, Phytochemistry and Pharmacological Review

**DOI:** 10.3390/molecules31071211

**Published:** 2026-04-07

**Authors:** Elmira Kartbayeva, Gulnaz Seitimova, Dinara Satmbekova, Meruyert Mukhitdin, Elmira Kabdylkanova, Aliya Kipchakbayeva

**Affiliations:** 1 Department of Fundamental Medicine, Faculty of Medicine and Health, Al-Farabi Kazakh National University, Almaty 050040, Kazakhstan; e.kartbayeva@gmail.com (E.K.); dskanatovna@gmail.com (D.S.); elmirakabdylkanova94@gmail.com (E.K.); 2 Faculty of Chemistry and Chemical Technology, Al-Farabi Kazakh National University, Almaty 050040, Kazakhstan; gulnaz.seitimova@gmail.com (G.S.); aliya_k85@mail.ru (A.K.)

**Keywords:** *Cirsium arvense*, *Cirsium vulgare*, phytochemistry, extraction, biological activity, phenolic compounds, flavonoids, non-native plant, invasive populations

## Abstract

The genus *Cirsium* (family *Asteraceae*, subfamily *Carduoideae*) comprises more than 200 species distributed throughout the temperate regions of the Northern Hemisphere. In recent years, particular scientific interest has focused on *Cirsium arvense* (L.) Scop. (creeping thistle) and *Cirsium vulgare* (Savi) Ten. (spear thistle). These species are notable for their high content of secondary metabolites and broad biological activity. However, the available data on their phytochemical composition and biological potential remain fragmented. This information is methodologically diverse and scattered across different scientific disciplines, underscoring the need for systematic analysis. In this study, a comprehensive literature review was conducted. Sources included PubMed, Scopus, Web of Science, Google Scholar, and other online databases. The focus was on phytochemical composition and pharmacological activity. Both species contain a wide range of secondary metabolites. These include phenolic acids (chlorogenic, caffeic, and ferulic acids), flavonoids (luteolin, apigenin, kaempferol, quercetin), triterpenoids (lupeol, taraxerol), and phytosterols. *C. vulgare* generally has higher levels of chlorogenic acid and flavonoid glycosides. In contrast, *C. arvense* has a greater abundance of triterpenes and steroidal compounds. Pharmacological studies show antioxidant, antimicrobial, hepatoprotective, anti-inflammatory, and cytotoxic activities for both species. Overall, the available data indicate that *C. arvense* and *C. vulgare* are promising sources of biologically active compounds with diverse pharmacological potential. Although there are some limitations regarding standardization and the depth of preclinical and clinical validation, the obtained results confirm their relevance for further pharmacological and phytochemical research.

## 1. Introduction

The genus *Cirsium* (family *Asteraceae*, subfamily *Carduoideae*) includes more than 200 species found in the temperate regions of the Northern Hemisphere [1]. Members of this genus *Cirsium* have medicinal properties and are used in traditional East Asian medicine [2,3,4]. In recent years, particular scientific interest has focused on *Cirsium arvense* (L.) Scop. (creeping thistle) and *Cirsium vulgare* (Savi) Ten. (spear thistle), which are characterized by a high content of secondary metabolites and a wide range of biological activities [5]. Despite their status as invasive and difficult-to-eradicate weeds, both are now valued as sources of phytochemicals for pharmaceutical and biotechnological uses.

The *C. arvense* is a perennial with a well-developed vegetative system and a strong capacity for vegetative reproduction. This explains its broad distribution and high ecological resilience. Phytochemical investigations have found triterpenoids (α- and β-amyrin, taraxasterol, lupeol), sterols (stigmasterol, β-sitosterol), phenolic acids, and flavonoids. Together, these explain the antioxidant, anti-inflammatory, and antimicrobial properties of this species. Additionally, *C. arvense* is considered a promising source of natural compounds with potential applications in the pharmaceutical and cosmetic industries [6,7].

In contrast, *C. vulgare* is a biennial spiny plant rich in phenolic compounds, such as chlorogenic, caffeic, and ferulic acids, as well as flavonoids (apigenin, luteolin, quercetin) and their glycosides. Because of its high antioxidant and polyphenol content, this species shows marked hepatoprotective, antitumor, and gastroprotective effects in experimental studies. As a result, *C. vulgare* extracts are being investigated as plant sources for antioxidant and anti-inflammatory phytopharmaceuticals [8,9,10,11,12,13].

For *C. arvense*, the available evidence is limited and mainly demonstrates antioxidant and antimicrobial activity. In contrast, *C. vulgare* has been reported to exhibit a wider range of experimentally confirmed activities, including hepatoprotective, gastroprotective, and cytotoxic [14]. Despite the growing number of publications, the available data remain methodologically heterogeneous. Studies often differ in the plant parts used, extraction procedures, analytical techniques, and experimental models. Such variability complicates comparisons between studies and limits a comprehensive evaluation of the pharmacological potential of these species. Moreover, studies integrating the phytochemical composition, biological activity, and traditional uses of these species from a modern, evidence-based phytopharmacological perspective remain scarce.

Many studies have investigated species of the genus *Cirsium*. However, the available information on *C. arvense* and *C. vulgare* remains scattered across different types of studies. Most publications focus on individual aspects, such as phytochemical constituents or specific pharmacological effects. Direct comparisons between these two species are still limited. As a result, the overall picture of their phytochemical diversity and biological potential is not clearly summarized in the literature. To the best of our knowledge, the present review represents the first comprehensive synthesis of data on *C. arvense* and *C. vulgare*. It summarizes their ethnobotanical uses, phytochemical composition, and pharmacological properties. In addition, the review discusses methodological differences between studies. The aim of this work is to organize the available evidence and to highlight directions for future preclinical and clinical research.

## 2. Methods

The literature describing the phytochemical composition, biological activity, and pharmacological properties of plants of the genus *Cirsium*, with particular emphasis on *C. arvense* and *C. vulgare*, was comprehensively reviewed. The review was performed following the general principles of the PRISMA 2020 guidelines to ensure transparency and reproducibility of the study selection process.

Data on *C. arvense* and *C. vulgare* were collected using databases such as PubMed, Scopus, Web of Science Core Collection, and Google Scholar, covering the period from the inception of database indexing to 15 January 2025. Publications in English and Russian were included. 

The search strategy employed the following keywords, in combinations, related to *Cirsium*, its species names, and associated phytochemical and pharmacological activities: antioxidant, antimicrobial, anti-tumor, anti-inflammatory, and hepatoprotective. For example, in PubMed the following search query was used: (*Cirsium* OR “*Cirsium arvense*” OR “*Cirsium vulgare*”) AND (phytochem* OR secondary metabolite* OR biological activity OR pharmacological property). In addition, taxonomic synonyms and alternative botanical names reported for these species (e.g., *Carduus arvensis* L. and *Carduus lanceolatus* L.) were considered during the search to minimize the risk of omitting relevant studies.

Studies were considered eligible if they clearly identified the plant species (*C. arvense or C. vulgare*), specified the plant part used, described extraction methods and solvents, and reported phytochemical composition and/or pharmacological activity using recognized analytical techniques such as GC–MS, HPLC, LC–MS, NMR, or spectrophotometry. Studies were excluded if the plant species was not clearly identified, if phytochemical or pharmacological data were insufficient, or if the full text was not accessible. 

Study selection was performed independently by two reviewers. Titles and abstracts were initially screened, followed by full-text evaluation of potentially relevant articles. Any disagreements were resolved through discussion and re-examination of the original publications. 

Data extraction was also performed independently by two reviewers. For each included study, information was collected on plant species and plant part; extraction method and solvent; identified compounds with concentrations (if applicable); analytical methods; type of biological assay; IC_50_/EC_50_ values; in vitro or in vivo model; botanical characteristics; geographical distribution; and traditional uses. No assumptions were made regarding missing or unclear data. 

**Figure 1:**
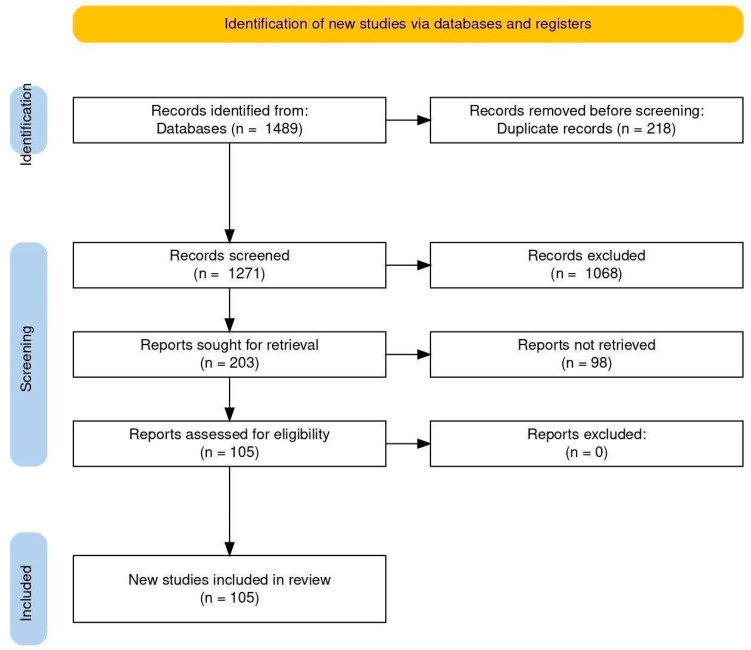
The PRISMA model.

Initially, 1489 publications were identified. After removing 218 duplicates, 1271 titles and abstracts were screened. Of these, 1068 records were excluded. A total of 203 articles were assessed for eligibility. Ninety-eight articles were excluded for insufficient data. Finally, 105 studies met the inclusion criteria and were included in the review. The PRISMA selection flowchart is shown in Figure 1. Excluded studies were mainly omitted due to insufficient phytochemical characterization or unclear species identification. 

Phytochemical characterization in the included studies was generally performed with solvent extracts of plant material, most often butanolic extracts. These extracts were analyzed using chromatographic techniques, such as GC–MS or HPLC, to identify major classes of secondary metabolites. However, analytical conditions varied considerably across studies, potentially affecting the comparability of phytochemical data.

To supplement these data, relevant background information from reviews and related studies (including general aspects of the genus *Cirsium* and broader topics such as flavonoids, triterpenes, structure–activity relationships, and nanomaterial-based applications) was also included, providing contextual support for findings specific to *C. arvense* and *C. vulgare*. All such references were included within the final set of 105 studies and were used to strengthen the interpretation and discussion of the findings.

This review was not registered and no protocol was prepared. No standardized risk of bias assessment tool was applied due to the heterogeneity of the included studies; however, study quality was considered qualitatively during interpretation of results. As a meta-analysis was not performed due to heterogeneity, no effect measures were calculated; results were summarized narratively.

## 3. Results and Discussion

### 3.1. Geographic Distribution, Taxonomy and Botanical Characteristics of the Genus Cirsium

**Figure 2:**
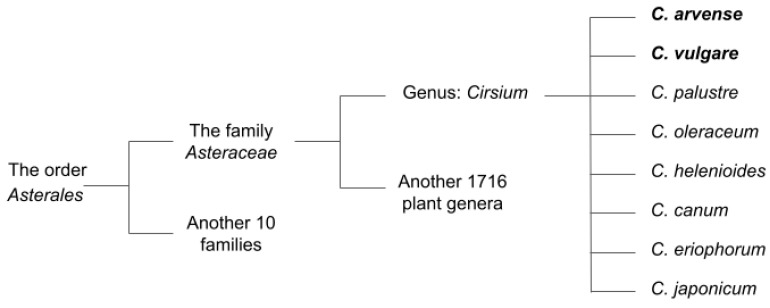
Most common species of the genus *Cirsium*.

The genus *Cirsium* comprises predominantly perennial and, less frequently, annual spiny plants belonging to the family Asteraceae (Figure 2), comprising approximately 1600 species and up to 23,000 species [15]. The genus name is derived from the Greek term khirsos, meaning “swollen vein” [16,17]. Representatives of *Cirsium* are distributed mainly across the Northern Hemisphere, including Europe, Asia, North America, and North Africa. Considerable diversity of the genus has been reported in East Asia, where approximately 120 species have been recorded in Japan and about 50 species in China [18,19,20]. Approximately 16 species are distributed in the humid evergreen forests of India and adjacent regions of China and Nepal, including *C. argyracanthum*, *C. arvense*, *C. lineare*, *C. eriophoroides*, *C. falconeri*, *C. flavisquamatum*, *C. interpositum*, *C. verutum*, *C. nishiokae*, *C. phulchokiense*, *C. shansiense*, *C. souliei*, *C. tibeticum*, *C. wallichii*, and *C. glabrifolium* [21]. Overall, representatives of the genus occur on all continents except Antarctica [22,23,24].

The genus *Cirsium* comprises herbaceous plants characterized by spiny leaves and purplish-pink capitulate inflorescences [25,26,27]. Most representatives of the *Asteraceae* family are perennial plants with a well-developed system of spines that may be present on all parts of the plant. Species of the genus *Cirsium* are commonly known as “thistles”, including creeping thistle (*C. arvense*) and spear thistle (*C. vulgare*) [28].

### 3.2. Botanical Characteristics and Distribution of Selected Species

**Figure 3:**
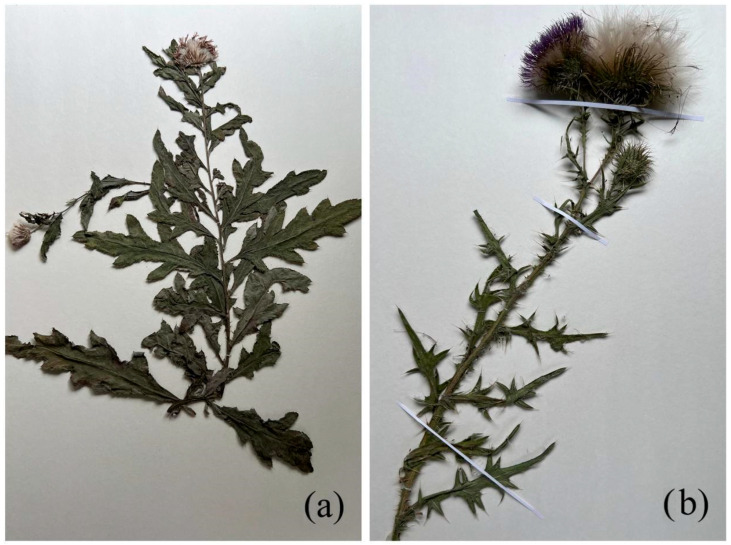
Herbarium specimens of (**a**) *C. arvense* and (**b**) *C. vulgare* deposited in the Herbarium of the Institute of Botany and Phytointroduction (AA), Almaty, Kazakhstan. Voucher information: voucher No. 9462. *C. arvense* collected in Almaty region, Ili district (43° 366385, E 76° 836954), 15 July 2025; *C. vulgare* collected in Almaty region, Enbekshikazakh district (43°16′20.6″, E 76°41′57.4″), 15 July 2025.

*C. arvense*—creeping thistle (Figure 3a). *C. arvense* is a perennial herbaceous plant reaching up to 150 cm in height. The root system is strong and creeping, forming numerous underground shoots that facilitate vegetative propagation. The leaves are pinnately lobed with sharp spines along the margins; the stem is erect and branched. The inflorescences are capitula 1–2 cm in diameter, arranged in corymbose panicles, with purple to violet florets.

To illustrate the morphological characteristics of *Cirsium arvense* (L.) Scop. and *Cirsium vulgare* (Savi) Ten., photographs of herbarium specimens deposited in the Herbarium of the Institute of Botany and Phytointroduction (AA), Almaty, Kazakhstan, were used (Figure 3a,b). Herbarium material enables visual comparison of plants from different sources and supports reliable taxonomic identification, which is particularly important in review studies, where accurate classification directly affects the interpretation of data on biological activity.

**Figure 4:**
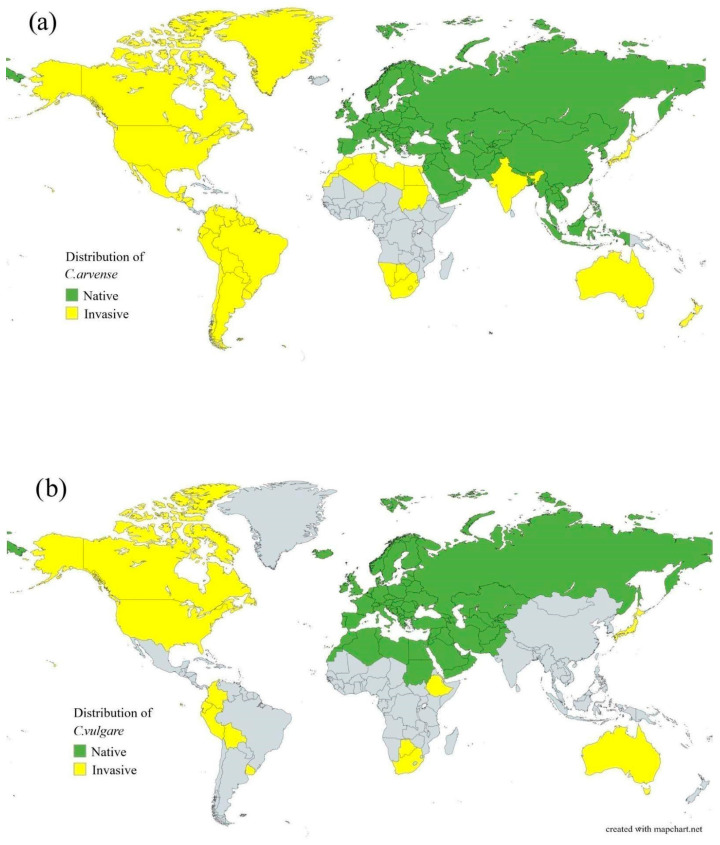
Global distribution of (**a**) *C. arvense* and (**b**) *C. vulgare*.

The species is widely distributed across Europe and Asia, and is considered invasive in North America, Japan, the Middle East, India, Australia, South America, New Zealand, and both Southern and Northern Africa (Figure 4a,b) [5,29,30]. It typically inhabits both cultivated and uncultivated lands.

The *C. vulgare*—spear thistle (Figure 3b). *C. vulgare* is a biennial plant that can reach up to 180 cm in height. During the first year, it forms a basal rosette, while in the second year it develops an erect, spiny, and pubescent stem. The leaves are large and rough, with a tomentose underside and long, sharp spines along the veins. The flowers are purplish-pink and arranged in large capitula [31].

The *C. vulgare* is one of the most invasive species within the genus and is widely distributed in Europe, Western Asia, and North Africa. It has also become naturalized in North America, several South American countries, and Australia (Figure 3b) [32,33].

**Figure 5:**
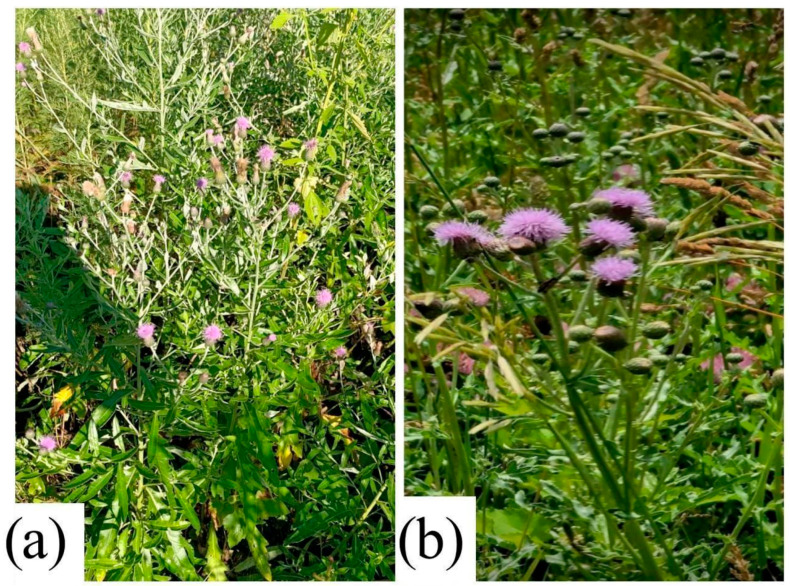
Natural habitats of *C. arvense* (**a**,**b**).

**Figure 6:**
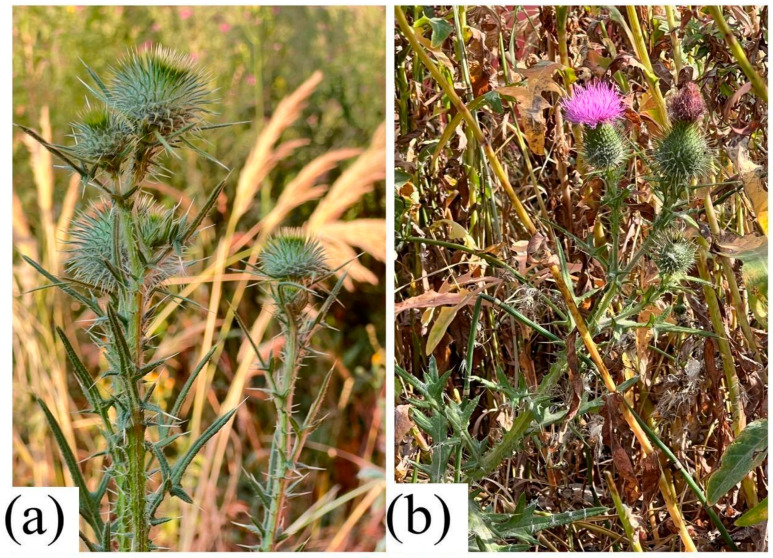
Natural habitats of *C. vulgare* (**a**,**b**).

Both species preferentially inhabit disturbed environments, including meadows, pastures, roadsides, and fallow lands. Their high ecological plasticity and pronounced ability for vegetative reproduction contribute to their wide distribution (Figure 5a,b and Figure 6a,b).

### 3.3. Traditional Uses

Medicinal plants are increasingly recognized as sources of new preclinical drugs. *Cirsium* species have been used in traditional medicine for hepatoprotective purposes and are also traditionally used to treat gastritis, diabetes, hemorrhoids, and cough [34].

The *C. arvense is* also used in traditional cuisine. Its roots provide inulin and starch, easily digestible carbohydrates. In some regions, the plant is eaten as a vegetable, salad ingredient, or seasoning. *C. arvense* also shows notable medicinal properties [35,36,37]. In China, hot infusions have been used to treat rheumatic diseases [38]. Decoctions have been used to treat bleeding, digestive disorders, hypertension, inflammation, scabies, ulcerative lesions, and skin diseases [39,40].

The *C. vulgare* has a long history of traditional use. In Poland, it serves as an anxiolytic, and in Polish popular medicine, it is also used as a diuretic, astringent, and anti-inflammatory remedy [29,33]. Meanwhile, in traditional Chinese medicine, *C. vulgare* is prepared as a decoction to treat inflammation, convulsions, and central nervous system (CNS) disorders [41].

### 3.4. Phytochemical Composition of C. arvense and C. vulgare

The *C. arvense* and *C. vulgare* possess a diverse phytochemical profile, featuring phenolic compounds, flavonoids, phenolic acids, lignans, triterpenes, sterols, and terpenoids [42,43]. Although classified within the same genus, the composition and relative abundance of bioactive constituents differ according to the plant part examined, the extraction solvent used, and the phenological stage at harvest [44]. In both species, phenolic acids—such as chlorogenic acid, caffeic acid, and p-coumaric acid—together with flavonoids like apigenin-7-O-glucoside, luteolin, kaempferol-3-O-glucoside, and quercetin-3-O-glucoside, prevail [2,45,46,47]. The major phytochemical groups reported in both species are listed in Table 1.

As summarized in Table 1, both species contain comparable classes of compounds; however, their relative concentrations diverge. *C. vulgare* exhibits elevated levels of phenolic acids and triterpenoids. In several studies, methanolic extracts of *C. vulgare* were found to contain substantial quantities of flavonoids and other phenolic constituents.

In contrast, *C. arvense* shows a higher proportion of triterpenes and phytosterols. Both species contain a complex array of phenolic compounds, flavonoids, triterpenoids, and sterols, although their relative abundances differ between the taxa.

#### 3.4.1. Flavonoids and Phenolic Acids

Flavonoids and phenolic acids are the major classes of phenolic compounds identified in species of the genus *Cirsium*. *C. arvense* contains phenolic contents of ~25 mg GAE/g of dry extract, which can be considered relatively low to moderate compared to other plant species. The flavonoids were found to have a content of ~22 mg QE/g of the dry extract [42]. This indicates that the phenolic content may vary depending on environmental conditions, extraction methods, and plant parts. Notably, the identification of individual phenolic compounds revealed the presence of several biologically relevant constituents at relatively low concentrations, while other studies have reported significantly higher total phenolic contents in different extracts of *C. arvense*, highlighting considerable variability in phenolic composition.

Building on the overview above, detailed analyses identified several major phenolic compounds in the aerial parts of *C. arvense*. The extract contained several compounds, with contents expressed as percentage (*w*/*w*) of the dry extract: chlorogenic acid (0.15%), kaempferol-3-O-methyl ester (0.18%), quercetin (0.18%), quercetin-3-glucoside (0.14%), luteolin-7-O-β-d-glucopyranoside (0.2%), hispidulin-7-O-β-d-glucopyranoside (0.18%), luteolin (0.36%), apigenin (0.37%), and kaempferol (0.18%). Other flavonoids and phenolic acids appeared at lower concentrations [48].

In relation to the chemical composition described, Polish researchers studied five *Cirsium* species and observed a strong positive correlation between total phenolic content and antioxidant activity in methanolic extracts (R^2^ ≈ 0.95), whereas a lower correlation was found for ethyl acetate fractions (R^2^ ≈ 0.8) [29]. However, the original study does not provide details on the statistical model used for the correlation analysis or the exact number of samples used in the calculation.

The *C. vulgare* has a diverse profile of phenolic acids—neochlorogenic, ferulic, vanillic, chlorogenic, caffeic, and p-coumaric acids are notable, while gallic, ellagic, rosmarinic, protocatechuic, syringic, gentisic, and hydroxybenzoic acids are present at lower levels [33,49,50,51,52]. It also contains flavonoids, predominantly luteolin, kaempferol, and quercetin, along with their glycosides: apigenin-7-O-glucoside, luteolin-7-O-glucoside, kaempferol-3-O-glucoside, and quercetin-3-O-glucoside.

The highest concentration of phenolic compounds was observed in extracts obtained from leaves collected at the end of the dormancy period, with concentrations ranging from 102.5 to 146.3 mg/g dry weight for chlorogenic acid, while apigenin-7-O-glucoside ranged from 6.9 to 27.6 mg/g [53].

#### 3.4.2. Terpenoids, Triterpenes, and Volatile Components

The genus *Cirsium* is characterized by a diverse profile of terpenoid compounds, including non-volatile triterpenes, sterols, and volatile constituents identified by GC–MS analysis.

In *C. arvense*, GC–MS analysis of the methanolic extract identified several major triterpenoid and sterol compounds, including olean-12-en-3-ol acetate (3β) (63.87%), lanosta-8,24-dien-3-ol acetate (3β) (12.12%), β-amyrin (6.19%), γ-sitosterol (6.09%), α-amyrin (5.24%), stigmasterol (3.29%), and carbonic acid 2-ethylhexyl heptadecyl ester (3.16%). Additional constituents reported in *C. arvense* include triterpenoids (α- and β-amyrin), phytosterols such as stigmasterol and γ-sitosterol, and saponins, alkaloids, and choline [54,55].

In *C. vulgare*, lupeol and its derivatives are the predominant triterpenoids [51,52,56]. Gas chromatographic analysis of the hexane extract revealed that 52% of its composition consists of terpenoids, predominantly triterpenes and their esters. The most abundant compounds are lup-20(29)-en-3-yl acetate (29.9%), lupeol (13.2%), and norolean-12-ene (5.2%). Analysis of both species shows a complex triterpenoid composition, with differences in the types of compounds and their relative abundances [57,58].

Volatile constituents in *Cirsium* species have also been characterized. A study conducted in Kazakhstan investigated the aerial parts of *C. arvense*. Specifically, GC-MS analysis of the extracts revealed that the volatile fraction consisted mainly of hydrocarbons, terpenes, sesquiterpenes, aldehydes, and organic acids and their esters [59]. Similarly, in *C. vulgare*, 24 volatile compounds have been identified, including tridecanoic acid, docosahexaenoic acid, methyl tetradecanoate, citronellol, and 2,3,5,6-tetramethylphenol [60,61]. The essential oil contains volatile terpenes and fatty acids, which are also present in the seeds of this plant and in other *Cirsium* species [62].

Further GC–MS analysis of the hexane extract of *C. vulgare* revealed the presence of 41 bioactive constituents, with terpenoids accounting for 52.89% of the total extract. The most abundant compounds were lup-20(29)-en-3-yl acetate (29.9%), lupeol (13.2%), linolenic acid ethyl ester (6.4%), norolean-12-ene (5.2%), 1-nonadecene (4.2%), 9,12-octadecadien-1-ol (4.9%), hexadecanoic acid (3.2%), 1-tricosene (2.9%), and cycloeicosane (2.6%) [43]. The majority of these constituents belong to ketones, aldehydes, fatty acids, and aliphatic hydrocarbons, reflecting the plant’s essential oil and volatile fraction.

Overall, *C. arvense* and *C. vulgare* exhibit a complex and chemically diverse terpenoid profile. *C. arvense* is characterized by a higher diversity of triterpenoids and steroidal compounds, whereas *C. vulgare* shows a greater contribution of triterpenes and volatile constituents. These differences highlight the chemical diversity of the two species and may contribute to their distinct biological activities, as discussed in the following section.

### 3.5. Structure–Activity Relationships of Major Bioactive Compounds

The pharmacological activities of flavonoids and triterpenes depend on specific structural features. These features modulate how the compounds interact with biological targets. For flavonoids, antioxidant and related effects depend on the arrangement and number of hydroxyl groups, conjugation, and electron-delocalizing substituents. A conjugated C2–C3 double bond with a 4-oxo function in the C ring, and an ortho-dihydroxy (catechol) group in the B ring, supports electron delocalization. This stabilizes flavonoid radicals during free-radical scavenging and enhances antioxidant activity. Substitutions at C5 and C7 on the A ring can also facilitate hydrogen atom transfer and increase activity. Methoxy substitution may modulate anti-inflammatory effects. Many studies have established these structure–activity relationships and their influence on free-radical scavenging both in vitro and in biological systems [63].

In *Cirsium* species, this is observed in flavonoids such as luteolin, apigenin, and kaempferol, as well as their glycosides, which share many of these structural features and contribute to antioxidant and cytoprotective activities.

Additionally, triterpenoids such as lupeol and taraxasterol are another major class of bioactive compounds in *Cirsium*. These pentacyclic structures have anti-inflammatory, antioxidant, and antineoplastic activity. Hydroxyl (–OH) and carboxyl (–COOH) groups in oleanane and ursane triterpenoids are critical for their interactions with pro-inflammatory mediators and signaling pathways [64,65].

### 3.6. Influence of Extraction Methods

The yield and composition of phenolic compounds in *Cirsium* species are strongly influenced by the extraction method. For *C. vulgare*, ultrasound-assisted extraction with 50% ethanol is optimal, yielding the highest levels of apigenin-7-O-glucoside and chlorogenic acid. Similarly, another study found that 50% ethanol was the most effective for extracting phenolics from *C. vulgare* leaves [66,67]. While these compounds are not unique to *Cirsium*, their abundance and consistency in *C. vulgare* extracts make them useful markers for phytochemical characterization and quality control of this species.

A study on the antioxidant activity of five *Cirsium* species conducted in Poland reported high antioxidant activity in extracts rich in phenolic compounds, suggesting a possible relationship between phenolic content and antioxidant effects.

In *C. arvense*, antioxidant content was highest with methanol and butanol extraction [68]. In *C. vulgare*, major phenolic compounds such as chlorogenic acid and apigenin-7-O-glucoside peaked during budding and flowering, highlighting the importance of the vegetative stage for standardization [69].

The highest total flavonoid content (25.73 mg CE/g) was found in methanolic extracts [34,38]. Alcohols like methanol and ethanol are effective due to the polarity of phenolic compounds. Methanol extracts polar phenolics well, while diethyl ether is best for volatile oils and lipophilic flavonoids, often in combination with other solvents [70,71]. However, the high toxicity of methanol and diethyl ether encourages the use of safer alternatives such as ethanol, water, or their mixtures. Ethanol is less toxic but mainly used for specific compound classes rather than as a general extraction solvent [72].

Extraction parameters, such as temperature and duration, are important. Ultrasound-assisted extraction improves solvent penetration, allowing for lower temperatures and shorter times. Most phenolic compounds (except chlorogenic acid) are extracted within 30 min of ultrasonication, whereas maceration at room temperature can take up to 48 h to yield apigenin-7-O-glucoside [3,34,67]. *C. arvense* is expected to follow similar patterns due to its phenolic and solubility profiles.

### 3.7. Activities of Cirsium Species: Biological and Pharmacological

The biological and pharmacological activity of representatives of the genus *Cirsium* is closely associated with their rich phytochemical composition. The principal biological effects include antioxidant, antimicrobial, antitumor, anti-inflammatory, hepatoprotective, and gastroprotective activities [69,73,74]. The main pharmacological activities, as well as other biological activities such as antioxidant effects, reported for both species are discussed below.

#### 3.7.1. Antioxidant Activity

Antioxidant activity represents one of the most extensively studied aspects of the genus *Cirsium*. Extracts of *C. arvense* have demonstrated a high capacity for scavenging DPPH and ABTS radicals, as well as significant reducing power in the FRAP assay [38]. The highest activity was observed in the methanolic and ethanolic extracts of leaves and flowers, which correlate with elevated levels of chlorogenic acid and luteolin-7-O-glucoside, compounds also characteristic of *C. vulgare* [2,75]. Similar results have been reported for *C. vulgare*. Methanolic leaf extracts show strong antioxidant activity. They reach 86.4% inhibition at 0.5 mg/mL [76]. Leaves generally exhibit higher antioxidant capacity than stems or inflorescences. This is due to differences in phenolic content [77]. Extracts from leaves collected at the end of the dormant phenological stage showed the highest antioxidant activity.

In addition, *C. vulgare* extracts significantly inhibit lipid peroxidation in hydrogen peroxide–induced models, suggesting a potential role in protecting cellular membranes from oxidative damage [78]. In another study, the antioxidant activity of a hydroalcoholic extract of *C. arvense* was assessed using the DPPH assay. The extract showed IC_50_ values of 436.4 µg/mL after 30 min and 750.2 µg/mL after 24 h of incubation. Under the same conditions, ascorbic acid was used as the reference antioxidant. It exhibited IC_50_ values of 143.72 and 187.4 µg/mL, respectively [43].

#### 3.7.2. Antimicrobial Activity

Several studies report antimicrobial activity of extracts from *C. arvense* and *C. vulgare* against both Gram-positive and Gram-negative microorganisms. A key finding is the bacteriostatic effect observed against strains such as *Staphylococcus aureus* and *Bacillus subtilis* [79].

Extracts of *C. arvense* inhibited the growth of *S. aureus*, *B. subtilis*, *Escherichia coli*, and *Candida albicans*, with inhibition zone diameters ranging from 8 to 19 mm [80]. Furthermore, *C. arvense* has also been shown to act as an adjuvant to antibiotics. Specifically, when combined with cefixime, extracts of *C. arvense* showed increased antibacterial activity against resistant strains, including MRSA, *Acinetobacter baumannii*, and *E. coli*. Synergistic effects were confirmed using checkerboard assays and time-kill kinetics. Additionally, protein-leakage analysis suggested that the combination may enhance bacterial membrane disruption [81].

For *C. vulgare*, pronounced activity has been reported against *S. aureus*, *E. coli*, and *Klebsiella pneumoniae*, with inhibition zones reaching up to 22 mm at 1 mg/mL [82]. Studies of non-polar fractions of *C. vulgare* have demonstrated significant content of terpenoids, sterols and fatty acids, which contribute to pronounced antimicrobial activity. Petroleum ether extracts exhibited inhibitory effects against *E. coli* and *S. aureus*, supporting the potential of this species as a source of natural antiseptic agents [34].

Antimicrobial activity was observed for ethanolic extracts against *S. aureus*, *Staphylococcus epidermidis*, *Pseudomonas aeruginosa*, *Proteus vulgaris*, and *C. albicans*, with minimum inhibitory concentrations (MICs) ranging from 8.35 to 16.7 mg/mL. These MIC values indicate relatively weak antimicrobial activity, suggesting limited potency of the extracts. In comparison, other extracts reported in this subsection exhibited inhibition zones ranging from 8 to 22 mm, reflecting varying degrees of antibacterial efficacy.

Additionally, apigenin-7-O-glucoside has demonstrated antibacterial activity, particularly against *S. aureus* and *Enterococcus faecalis* [27,67,83].

In comparative analyses, aqueous and alcoholic extracts were evaluated, and the results revealed that alcoholic extracts are more effective against Gram-positive bacteria, whereas aqueous extracts generally exhibit weaker antimicrobial activity [84].

#### 3.7.3. Antitumor Activity

Both in vitro and in vivo studies have demonstrated cytotoxic activity of *Cirsium* extracts against several cancer cell lines [85].

In a recent study by Griškevičienė et al. (2025) [86], dry extracts from various plant parts of *C. vulgare* at different growth stages exhibited cytotoxicity against gastric cancer (KATO III) and colorectal cancer (HT-29) cell lines. The highest activity was observed for extracts derived from inflorescences and roots, with IC_50_ values of approximately 0.19 mg/mL and 0.35 mg/mL, respectively. In addition, these extracts significantly inhibited cell migration at 0.2 mg/mL and reduced spheroid viability by approximately 77–81%. Although these values indicate measurable anticancer potential, they remain relatively high compared with those typically reported for purified anticancer agents and therefore suggest moderate cytotoxicity of the crude extracts [86].

Dose-dependent cytotoxic effects of *C. vulgare* extracts have also been reported against breast carcinoma (MCF-7), colorectal carcinoma (HT-29), and hepatocellular carcinoma (HepG2) cell lines [87]. Similar antiproliferative activity of *C. arvense* extracts has also been reported against several cancer cell lines, including HeLa and C6 cells. In BrdU-ELISA assays, different solvent fractions inhibited cell proliferation, with the strongest effect observed in HeLa cells [88].

#### 3.7.4. Anti-Inflammatory Activity

The anti-inflammatory effects of *C. arvense* and *C. vulgare* extracts are mainly attributed to their flavonoid- and phenolic-rich composition. Experimental studies on other *Cirsium* species have demonstrated inhibition of nitric oxide production and modulation of inflammatory pathways involving COX and LOX enzymes [89].

In experimental models, *C. arvense* extract administered at a dose of 200 mg/kg reduced carrageenan-induced paw edema in rats by 49% [90]. Studies on *C. vulgare* have demonstrated suppression of nitric oxide and prostaglandin production in RAW 264.7 macrophages. Furthermore, *C. vulgare* extracts reduced nitric oxide levels and the production of pro-inflammatory cytokines in lipopolysaccharide-activated macrophages, indicating inhibition of the NF-κB signaling pathway and, consequently, pronounced anti-inflammatory activity [91].

#### 3.7.5. Hepatoprotective and Gastroprotective Activities

Extracts of *C. vulgare* exhibit pronounced hepatoprotective properties. In an experimental model of carbon tetrachloride (CCl4)-induced hepatitis, administration of *C. vulgare* extract at 200 mg/kg significantly reduced serum ALT and AST levels and restored liver histology. Hepatoprotective effects have also been reported for nonpolar (hexane) extracts of *C. vulgare* inflorescences in rat models of acute liver injury. These effects have been associated with phenolic compounds such as chlorogenic acid and luteolin, as well as triterpenoids including lupeol derivatives [61,92].

Moderate hepatoprotective activity has also been reported for *C. arvense*; however, available data are limited primarily to animal models [93]. Gastroprotective effects of *C. vulgare* have also been reported. Extracts reduced ethanol-induced gastric ulcer formation, likely due to increased secretion of the gastric mucus barrier [94].

#### 3.7.6. Other Pharmacological Activities

Both species have been reported to exhibit diuretic, hypoglycemic, and neuroprotective effects [95]. Administration of *C. vulgare* extract significantly reduced blood glucose levels in rats with alloxan-induced diabetes [96]. Anticoagulant activity has also been reported for *C. arvense* extracts [97].

**Figure 7:**
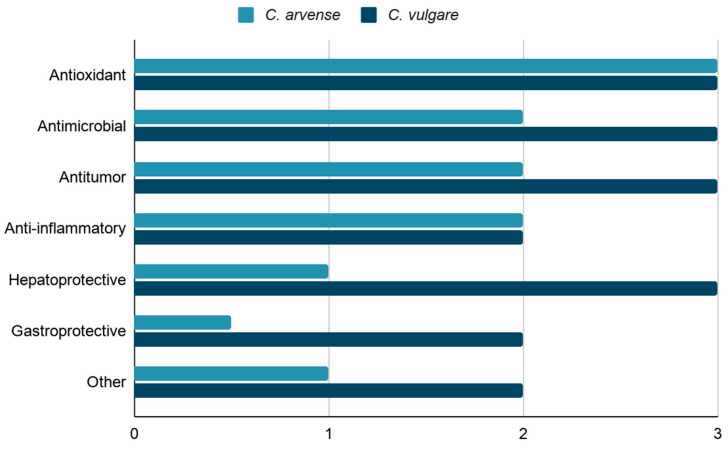
Comparison of pharmacological activities of *C. arvense* and *C. vulgare*. The numerical values indicate the relative level of literature support for each reported biological activity based on a semi-quantitative assessment of the number of studies, consistency of findings, and presence of associated bioactive constituents (1 = low, 2 = moderate, 3 = high) [34,38,77,80,82,83,84,85,86,87,88,89,90,91,92,93,95,96,97].

Based on the available literature data on the phytochemical composition of *C. arvense* and *C. vulgare*, a comparative diagram of their reported pharmacological activities was constructed (Figure 7). The diagram summarizes the main biological effects associated with the major groups of bioactive compounds identified in both species. The relative activity levels shown in the figure were assigned using a semi-quantitative literature-based scoring approach. The scores reflect the relative degree of published support for each activity, considering the number of studies, consistency of findings, and the occurrence of bioactive compounds associated with the corresponding effects. In this scale, 1 indicates low, 2 moderate, and 3 high relative support in the literature.

Overall, *C. vulgare* exhibits a broader and more pronounced spectrum of biological activities, whereas *C. arvense* shows a narrower but well-defined profile, with particularly strong antioxidant properties.

#### 3.7.7. Toxicological and Pharmacokinetic Aspects

Limited toxicological data are available for *C. arvense* and *C. vulgare*. One recent study found that ethanol extracts of *C. arvense* aerial parts were toxic to brine shrimp (*Artemia salina*) with an LC_50_ of 51 μg/mL, showing cytotoxicity at high in vitro concentrations [98]. In vitro studies show that *C. vulgare* flower extracts are usually more bioactive than leaf extracts. Methanol-water-trifluoroacetic acid (50:50:0.1) extracts of *C. oleraceum* (flowers) and *C. vulgare* (flowers and leaves) showed low cytotoxicity at 1.25 mg/mL, with cell viabilities of 92.6 ± 8.9%, 82.2 ± 13.1%, and 87.5 ± 7.9%, respectively [99]. No comprehensive studies have assessed acute, subchronic, or chronic effects, reproductive effects, or genotoxic effects in animal models, and clinical safety data are lacking. Pharmacokinetic data for *C. arvense* and *C. vulgare* extracts are also lacking. Most available data come from related species, such as *Cirsium japonicum*, in which flavonoids were quantified in rat plasma after oral administration, providing preliminary insights into absorption and systemic exposure [100].

#### 3.7.8. Nanoparticles

Both species are also being explored as potential candidates for the biosynthesis of metal nanoparticles. For example, plant extracts of *C. arvense* have been used for the green synthesis of silver nanoparticles, which demonstrated antibacterial activity against *Escherichia coli* [101,102]. In addition, copper nanoparticles synthesized using *C. arvense* extracts have been reported to exhibit photocatalytic activity and antibacterial effects against *Staphylococcus aureus* and *E. coli*, with inhibition zones of approximately 18 and 21 mm, respectively [103,104]. Extracts of *C. vulgare* have also been used to biosynthesize cobalt oxide nanoparticles with promising electrocatalytic properties [105].

## 4. Comparative Analysis and Discussion

The *C. arvense* and *C. vulgare* share broadly similar phytochemical profiles and biological properties. Both species contain phenolic compounds, such as chlorogenic and caffeic acids, as well as flavonoids, including luteolin, apigenin, kaempferol, and quercetin, and their glycosides. These compounds are considered major contributors to the antioxidant activity reported for *Cirsium* species.

A critical factor influencing experimental outcomes is the methodology used for preparing plant material. The composition of extracts and the magnitude of biological activity depend strongly on the plant part analyzed, the phenological stage at harvest, the solvent type, and the extraction technique used (e.g., maceration, ultrasound-assisted extraction, or reflux extraction). This methodological variability complicates the direct comparison of results across different studies.

Despite these shared features, the two species differ markedly in the dominant classes of compounds and in the extent of their pharmacological investigation. *C. vulgare* is characterized by higher concentrations of phenolic acids, particularly chlorogenic acid and apigenin-7-O-glucoside. In contrast, *C. arvense* contains higher levels of triterpenes and sterols, including α- and β-amyrin, taraxasterol, and stigmasterol. Although both species have been investigated for various biological and pharmacological activities, more pharmacological studies have been reported for *C. vulgare* than for *C. arvense*.

Some relationship between phytochemical composition and biological activity can also be considered. Phenolic acids and flavonoids reported in *Cirsium* species, such as chlorogenic acid, luteolin, and apigenin derivatives, are commonly associated with antioxidant and anti-inflammatory activities. In contrast, triterpenoid constituents, including lupeol and amyrin derivatives, are more often discussed in relation to hepatoprotective and cytotoxic effects observed in experimental models. Regional and phenological variability also differ between the species. For *C. vulgare*, a clear dependence of marker compound content on geographic origin and developmental stage has been demonstrated, creating favorable conditions for standardizing plant raw materials. Comparable data for *C. arvense* remain scarce and less detailed. From a practical perspective, these differences suggest that *C. vulgare*, owing to its high lupeol and chlorogenic acid content, may be a promising candidate for hepatoprotective applications, whereas *C. arvense*, characterized by the pronounced antimicrobial activity of specific fractions, may be a potential source of antibacterial compounds and natural antiseptics. Recent studies also suggest that the *Cirsium* species may represent promising biological sources for the green synthesis of metal nanoparticles, although this field remains at an early experimental stage.

### 4.1. Limitations of Current Research

Contemporary research highlights several critical knowledge gaps. In addition, the overall quality of the available evidence varies considerably. Many studies rely mainly on in vitro assays, while well-controlled in vivo investigations remain relatively limited. Differences in extract preparation, plant material origin, and experimental models further complicate direct comparison between studies. Moreover, the absence of standardized extracts and consistent dose reporting often restricts the interpretation and translational relevance of the reported biological effects.

The most prominent limitation is the lack of standardized extraction protocols: key parameters, such as plant-to-solvent ratio, extraction temperature, and extraction time, are often insufficiently reported, severely limiting data comparability. The volume of preclinical research remains limited, and only a small number of studies provide detailed pharmacodynamic data for *Cirsium* extracts, while information on pharmacokinetics and bioavailability is largely lacking. Although phenolic compounds commonly reported in *Cirsium* extracts, such as chlorogenic acid, luteolin, and apigenin derivatives, are known to undergo rapid metabolism, specific pharmacokinetic studies evaluating these metabolites in extracts of *C. arvense* or *C. vulgare* in animal models or humans have not yet been reported. Most available studies focus primarily on in vitro biological activity and phytochemical profiling, whereas data on absorption, metabolism, and systemic availability of the main constituents remain scarce.

Comprehensive toxicological evaluations, including acute, chronic, and reproductive toxicity, are largely absent. Furthermore, these species are considered invasive weeds and are therefore frequently treated with herbicides. The possible presence of pesticide residues in wild-collected plant material represents an additional safety concern and may limit their use in pharmaceutical applications. Moreover, molecular mechanisms of action remain insufficiently elucidated; the involvement of key signaling pathways, such as MAPK, NF-κB, Nrf2, and annexin A2, has been addressed only sporadically. Clinical trials (Phases I–III) are entirely lacking, precluding any robust assessment of the efficacy and safety of *Cirsium*-based herbal preparations. Additionally, regional data—particularly from Central Asia—remain scarce, despite the wide distribution of both species in this region.

The present review has several limitations related to the review process. No meta-analysis was conducted due to methodological heterogeneity, and a formal risk of bias assessment was not performed. In addition, the synthesis was narrative in nature, which may introduce interpretative subjectivity.

### 4.2. Future Research Perspectives

Promising directions for future research include standardizing and validating extraction methodologies, identifying and implementing reliable chemical markers (e.g., chlorogenic acid and apigenin-7-O-glucoside for *C. vulgare* and α-amyrin for *C. arvense*), and conducting comprehensive preclinical studies that incorporate pharmacodynamic, pharmacokinetic, and toxicological profiling. Further investigation into the molecular mechanisms of action of bioactive compounds is also warranted, particularly their effects on key pathways involved in apoptosis, inflammation, and oxidative stress, as well as their interactions with living tissues and mechanisms underlying antimicrobial activity. Additional emphasis should be placed on evaluating the synergistic potential of *Cirsium* extracts in combination with antibiotics and on initiating pilot clinical studies to assess safety and efficacy for specific preclinical indications. In Central Asian regions, a systematic evaluation of the impact of climatic and geographical factors on marker compound content and raw material quality is an important and timely research objective.

## 5. Conclusions

Despite a significant number of available publications, most research on *C. arvense* and *C. vulgare* remains limited to laboratory studies, and large-scale preclinical and clinical trials are lacking. The main limitations include the lack of standardized extraction and analytical methods and insufficient understanding of the relationships between the content of individual phenolic compounds and specific biological effects. Most data on *C. arvense* demonstrate primarily antioxidant and antimicrobial activities. *C. vulgare*, in contrast, has been more extensively investigated in preclinical studies and may have additional hepatoprotective, gastroprotective, and cytotoxic potential. Comprehensive studies combining phytochemical, pharmacological, toxicological, and clinical approaches will be crucial for future research.

## Figures and Tables

**Table 1:** Major phytochemical groups identified in *C. arvense* and *C. vulgare*.

Compound Group	Main Representatives	Plant Parts	*C. arvense*	*C. vulgare*
Flavonoids [22,48,49,50]	Apigenin, Luteolin, Kaempferol, Quercetin	Leaves and flowers (including inflorescences)	+	+
Flavonoid glycosides [45,46,47,49]	Apigenin-7-O-glucoside, Luteolin-7-O-glucoside, Kaempferol-3-O-glucoside, Quercetin-3-O-glucoside	Leaves and flowers	+	+
Phenolic acids [45,46,47,51,52,53]	Chlorogenic, Caffeic, p-Coumaric	Leaves and flowers (including inflorescences)	++	+++
Triterpenoids [24,54]	Lupeol	Flowers, roots, herbs	+	++
Other compounds [42,43,54]	Phytosterols, Saponins, Alkaloids, Choline, Citronellol, Scopoletin, Lignans	Various parts	±	±

Note: “+”—presence of compounds; “++”—high concentration; “+++”—dominant group; “±”—sporadic detection in the literature.

## Data Availability

No new data were created or analyzed in this study. Data sharing is not applicable to this article. All data analyzed in this study are included in the published article.

## Abbreviations

The following abbreviations are used in this manuscript:

CGA	chlorogenic acid
A7G	apigenin-7-O-glucoside
ABTS	2,2′-Azino-bis(3-ethylbenzothiazoline-6-sulfonic acid)
DPPH	2,2-Diphenyl-1-picrylhydrazyl
GC–MS	Gas chromatography–mass spectrometry
HPLC	High-performance liquid chromatography
MRSA	Methicillin-resistant *Staphylococcus aureus*
RP-HPLC-DAD	Reversed-phase high-performance liquid chromatography with diode-array detection
MIC	minimum inhibitory concentration
COX	cyclooxygenase
LOX	lipoxygenase
IL-6	Interleukin-6
TNF-α	Tumor necrosis factor alpha
ALT	Alanine aminotransferase
AST	Aspartate aminotransferase
CCl_4_	Carbon tetrachloride
MAPK	Mitogen-activated protein kinase
NF-κB	Nuclear factor kappa B
Nrf2	Nuclear factor erythroid 2–related factor 2
